# Comparison between low cost USB nailfold capillaroscopy and videocapillaroscopy: a pilot study

**DOI:** 10.1093/rheumatology/keaa723

**Published:** 2020-11-24

**Authors:** Michael Berks, Graham Dinsdale, Elizabeth Marjanovic, Andrea Murray, Chris Taylor, Ariane L Herrick

**Affiliations:** 1 Centre for Imaging Sciences, Division of Informatics, Imaging & Data Sciences, The University of Manchester, Manchester, UK; 2 Division of Musculoskeletal and Dermatological Sciences, The University of Manchester, Salford Royal NHS Foundation Trust, Manchester Academic Health Science Centre, Manchester, UK

**Keywords:** SSc, nailfold capillaroscopy, videocapillaroscopy, USB microscope

## Abstract

**Objectives:**

Universal serial bus (USB) microscopy (capillaroscopy) could provide all rheumatologists with an easy-to-use, low-cost tool to examine the nailfold capillaries to facilitate early diagnosis of SSc. The objectives of this pilot study were to examine the feasibility of acquiring and analysing images using USB microscopy and to compare results to videocapillaroscopy.

**Methods:**

Videocapillaroscopy and USB microscope images were obtained from the right and left ring fingers of 20 patients with SSc and 20 healthy control subjects. In addition to generating panoramic capillary mosaics from across the whole nailbed, custom software made fully automated measurements of vessel structure including capillary width and density. The area under the receiver operating characteristic curve (A_Z_) was used to measure separation between the SSc and healthy control groups.

**Results:**

High quality images could be generated from the USB microscope, with reconstructed USB images comparing very favourably with those obtained using videocapillaroscopy. Using USB microscope images, the receiver operating characteristic curve A_Z_ for group separation based on mean width was 0.81 (standard error 0.120) compared with 0.81 (standard error 0.095) for the (gold standard) videocapillaroscopy. The receiver operating characteristic curve A_Z_ for group separation using capillary density was 0.48 (standard error 0.16) for USB microscope images, compared with 0.70 (standard error 0.10) for videocapillaroscopy.

**Conclusion:**

In this pilot study, USB capillaroscopy was able to discriminate between patients with SSc and controls as well as videocapillaroscopy on the basis of capillary width. This finding, together with the high-quality images obtained, highlights the potential of USB capillaroscopy as a low-cost, easily accessible clinical and research tool.

**Rheumatology key messages**

- High-quality nailfold capillary images can be obtained with USB capillaroscopy.
- In a pilot study, USB capillaroscopy compared favourably to videocapillaroscopy with respect to capillary width.
- USB capillaroscopy has potential as a low-cost, easily accessible clinical and research tool.

## Introduction

It is now well recognized that in the patient presenting with Raynaud’s phenomenon, abnormal nailfold capillaries are a ‘red flag’ and independent risk factor for progression to SSc [1–3]. This is acknowledged in the ACR/EULAR 2013 classification criteria for SSc [4, 5]: abnormal nailfold capillaries are one of the criteria. It therefore behoves all rheumatologists responsible for assessing patients with Raynaud’s phenomenon and/or diagnosing SSc-spectrum disorders to have access to nailfold capillaroscopy. Otherwise, a diagnosis of SSc could be missed, thus denying patients access to early intervention to prevent the development of painful digital ulcers, critical digital ischaemia and potentially life-threatening internal organ involvement. However, at present the majority of rheumatologists do not incorporate nailfold capillaroscopy into their everyday clinical practice, because capillaroscopy systems are not widely available, and the images once obtained can be difficult to interpret.

Several different techniques can be used to visualise the nailfold capillaries [6]. The gold standard technique is videocapillaroscopy (and is the one with which European rheumatologists with an interest in SSc are most familiar [7]), but this requires expensive equipment and it is unlikely that its use will become widespread among general rheumatologists. Low-cost hand-held microscopes can also visualise the nailfold capillaries; for example, dermoscopy compares favourably to nailfold videocapillaroscopy [8, 9].

Universal serial bus (USB) microscopy—using a digital microscope connected to a computer or tablet through a standard USB cable to record magnified video images—could be the answer to providing all rheumatologists with an easy-to-use, low-cost, hand-held tool to examine the nailfold capillaries. Yet, experience with USB capillaroscopy is limited, with no previous studies comparing it to other capillaroscopy systems. The aim of this pilot study was to examine the feasibility of acquiring and analysing images using the USB microscope and to compare results obtained to those from videocapillaroscopy images. Successful capture and quantitative analysis of nailfold capillary images from a USB microscope could bring quantitative (objective) nailfold capillary imaging within reach of the general rheumatology clinic.

## Patients and methods

### Patients

Twenty patients with limited cutaneous SSc were recruited in to the study (17 female, three male, median age 63 years, range 44–75, median duration of Raynaud’s phenomenon 21 years, range 4–42, median disease duration since the onset of first non-Raynaud’s manifestation 15 years, range 3–37) and 20 healthy controls, none of whom had Raynaud’s (15 female, five male, median age 52 years, range 25–60). All subjects attended for one visit when nailfold capillaroscopy was performed using both videocapillaroscopy and USB microscopy (see below) by a single technician. All were asked to abstain from smoking and from caffeine-containing drinks for four hours prior to the study visit. The study was approved by West of Scotland Research Ethics Service and all patients gave informed written consent.

### Methods

**Fig. 1:**
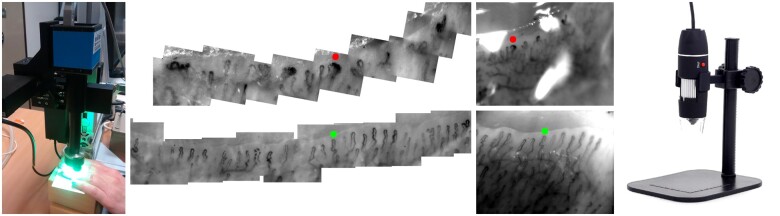
Videocapillaroscopy and USB nailfold capillaroscopy The videocapillaroscopy system (far left) and USB microscope (far right) with corresponding images (centre left: videocapillaroscopy, centre right: USB) from (upper) the same patient with SSc, and (lower) the same healthy control subject. Red and green markers highlight the same capillary in the two image types for patient and control images, respectively.

All subjects underwent a 20 min period of acclimatization at 23°C in a temperature and humidity controlled room. Nailfold capillaroscopy was performed of left and right ring fingers, using (Fig. 1): (i) high-precision, custom-built, videocapillaroscopy system, taken as the ‘gold standard’; (ii) a low cost, hand-held USB microscope. The imaging technician was not blinded to subjects’ disease status, but a strictly standardized imaging protocol was followed.

#### Videocapillaroscopy

The system used has been described in detail elsewhere [10, 11]. In brief, a high frame rate (120 frames per second) camera and lens system are mounted on a software-controlled three-axis motorized stage, allowing rapid capture of video sequences across the nailfold. From these video sequences, custom software generates high-quality nailfold capillary mosaics (Fig. 1) and then makes fully automated measurements of capillary structure and flow [11]. Image resolution is ∼1 µm/pixel.

#### USB capillaroscopy

This technique uses a commercially available, low-cost consumer device. In this study we used an XCSOURCE TE389 purchased via Amazon, although many similar devices are available from as little as $25. They contain a colour camera sensor similar to those found in common webcams, and plastic optics with zoom/focus controls, allowing high-magnification imaging. Final image resolution is ∼2–6µm/pixel.

#### Automated image analysis

We used a super-resolution method [12] to generate composite images from the USB microscope video data. For quantitative comparison between videocapillaroscopy and USB microscopy, we focused on mean capillary diameter (width) which was the single measure which best discriminated between subjects with and without SSc in our previous study [11] and capillary density (the measure with which rheumatologists are most familiar). To generate these (and other) parameters, the software first detects each distal row capillary and estimates the location of its apex. The average width of each capillary is computed from estimates of the width at each point along the capillary path up to a distance of 100 μm from the apex. For each nailfold image, the mean width was calculated (the mean of the individual capillary widths) and the capillary density (the number of capillary apices per millimetre, measured from the left-most to the right-most capillaries). For each participant, both of the nailfold-level measurements (i.e. right and left ring finger) were averaged to produce participant-level parameters.

#### Statistical analysis

Our analysis involved a combination of qualitative (descriptive) and quantitative methods. For a quantitative comparison, the area under the receiver operating characteristic curve (ROC A_Z_) was used to measure separation between the SSc and healthy control groups using the automated measurements of mean width and capillary density.

## Results

### Application of novel software to the USB images

**Fig. 2:**
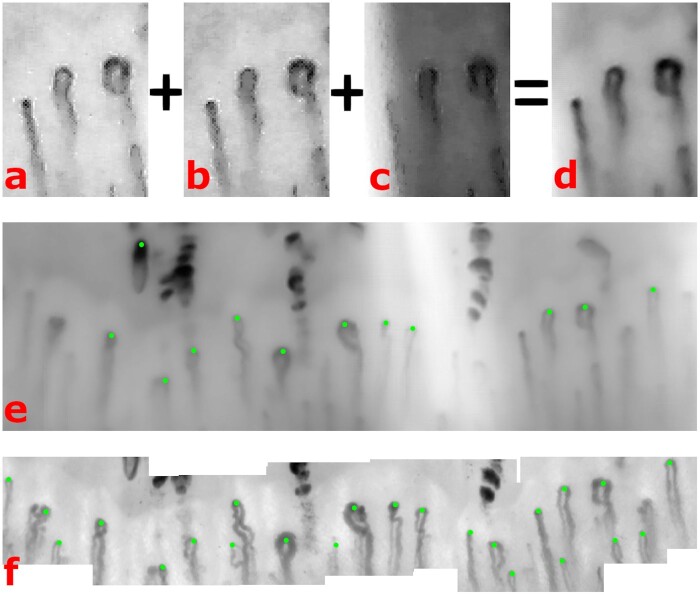
Imaging a nailfold with a USB microscope (**a**–**e**) compared with videocapillaroscopy (**f**). Images (**e**) and (**f**) show the mosaic reconstructed with multiple frames using the USB microsope and videocapillaroscopy respectively. For full explanation, see text.

Fig. 2 demonstrates that high quality images can be generated from the USB microscope. Fig. 2a–c show three video frames of the same section of a nailfold, captured using the USB microscope. Although capillaries are visible, they are not sufficiently well defined to allow measurement of morphology and in some frames they are obscured, for example by glare in Fig. 2c. Fig. 2d shows a ×4 super-resolution reconstruction of the same region using 15 frames in total. Fig. 2e shows the reconstruction of the whole nailbed obtained using the USB microscope, and Fig. 2f the reconstruction of the same nailbed using videocapillaroscopy. Green dots show capillaries detected by the analysis software. Taking videocapillaroscopy as ‘gold standard’, although USB results are not perfect (some apices have not been marked on the USB image), the majority of the apices have been identified, and to the naked eye the image quality (Fig. 2e) is very good and would easily allow the qualitative analysis [6, 13] that is the current ‘cornerstone’ of capillaroscopic analysis for most rheumatologists.

### Comparison of patients with SSc and healthy controls

#### Mean width

Mean width measured automatically in USB microscope images from healthy controls was 15.0 μm (95% CIs 12.5, 17.4), and in patients with SSc 31.2 μm (95% CI 23.7, 38.6), with area under the receiver operator curve (ROC A_Z_) 0.81 (standard error 0.120). With the videocapillaroscopy system, mean width in healthy controls was 12.1 μm (95% CI 11.4, 12.9), and in patients with SSc 19.0 μm (95% CI 16.1, 21.8), with area under the receiver operator curve (ROC A_Z_) 0.81 (standard error 0.095) (Supplementary Fig. S1, available at *Rheumatology* online).

#### Capillary density

Mean density using the USB microscope in healthy controls was 4.68/mm (95% CI 2.75, 6.61), and in patients with SSc 3.78/mm (95% CI 2.71, 4.68), with area under the receiver operator curve (ROC A_Z_) 0.48 (standard error 0.16). With the videocapillaroscopy system, mean density healthy controls was 6.43/mm (95% CI 5.19, 7.67), and in patients with SSc 3.99/mm (95% CI 3.12, 4.87), with area under the receiver operator curve (ROC A_Z_) 0.70 (standard error 0.10) (Supplementary Fig. S1, available at *Rheumatology* online).

## Discussion

The key findings from our study are that high quality nailfold capillaroscopy images can be obtained using USB capillaroscopy, and that (at least for capillary width measurements) the ability to discriminate between patients with SSc and healthy controls is as good using USB capillaroscopy as with videocapillaroscopy. Thus, there are implications for both clinicians and for researchers. For clinicians, the message is that it is well worthwhile buying a low-cost USB microscope for everyday clinical practice for assessment of patients presenting with Raynaud’s phenomenon or with other pointers to a SSc-spectrum disorder. For researchers, the ability to obtain high-quality images for quantitative (objective) assessment from low-cost systems opens up the vista of ‘big’ capillaroscopy data. Large-scale studies involving thousands of patients seen in different clinical settings now seem achievable because clinicians from both secondary and tertiary rheumatology centres across different continents could all have easy access to the necessary equipment (a USB microscope). Nailfold capillaroscopic findings have been suggested as both predictors and biomarkers of the SSc disease process [14, 15], and large prospective studies could examine predictors of disease progression and of treatment response with a degree of precision not previously possible. Automated image analysis [11] makes pooling images and data from large-scale studies feasible.

The limitations of our study include its small sample size: this was very much a pilot study to examine proof-of-concept and future studies should include larger patient numbers including those with diffuse cutaneous SSc. Also, we have yet to optimise the software for image acquisition and analysis using the USB microscope. The software was ‘trained’ on videocapillaroscopy images, and the learning does not transfer perfectly to images which, although good quality, have slightly different characteristics. This is most obvious for vessel detection, where the quantitative vessel density measurements, and qualitative comparison (see Fig. 2) indicate for the USB images the automated analysis missed a proportion of vessels detected in the videocapillaroscopy images. However, where capillaries were detected, clinically useful measures of capillary morphology were obtained. In further work, shortcomings in capillary detection may be addressed by: (i) acquiring raw video data at higher magnification (similarly low-cost USB microscopes with 2–3 times higher magnification are now available); (ii) refining the application of the super-resolution technique to capillary images; and (iii) applying transfer learning techniques [16] to refine the automated analysis to the specifics of USB microscopy data. In addition, the study design comparing devices only allowed for two fingers per device to be examined. Where a USB microscope is used as the sole device in a capillaroscopy examination, there is potential to analyse more fingers, which previous studies suggest would improve diagnostic accuracy [17].

Our study focused on automated, quantitative analysis. Studies comparing qualitative and semi-quantitatve analyses (e.g. ‘early’, ‘active’ and ‘late’ scleroderma patterns [6]) between USB microscopy and videocapillaroscopy would also be of interest.

It seems surprising that there has been so little research into low-cost capillaroscopy sytems, despite the increasing interest in nailfold capillaroscopy internationally. A recent pilot survey of 42 US clinicians with an interest in SSc [18] found that only 7% used videocapillaroscopy compared with 64% who used either a dermatoscope or opthalmoscope, suggesting that at least in the United States, the preference of SSc specialists is for lower cost hand-held systems (USB microscopy was not mentioned). Conversely, in a survey of 88 (mainly European) adult rheumatologists [7], 90.9% felt their knowledge of videocapillaroscopy was either good or satisfactory, whereas fewer than 40% felt similarly for USB microscopy. Therefore, clinical practice, at least in specialist centres, currently differs between countries.

We are not aware of previous reports comparing USB microscopy to videocapillaroscopy, although in 2012, Bhakuni *et al*. [19] highlighted the potential of USB microscopy to examine nailfold capillaries in a study including 42 patients with SSc and 42 control subjects. As earlier stated, dermoscopy has been compared with videocapillaroscopy. Dermoscopy (similarly to USB microscopy) is hand-held and therefore easily portable, but requires an attached camera for image capture and is more expensive (in the order of $1000–$2000) than a USB microcope. A recent study comparing nailfold capillary photographs taken with a ‘smartphone lens’ and with a ‘smartphone dermatoscope’ to widefield microscopy [20] highlighted the potential of low-cost, portable devices for use in routine clinical practice.

In conclusion, our findings indicate that USB capillaroscopy is a highly promising, low-cost technique that could well be the way forward for maximizing the potential of nailfold capillaroscopy as a diagnostic and research tool. Next steps are to raise awareness of the technique among rheumatologists and to refine automated analysis not only to improve diagnostic accuracy but to pave the way for large-scale capillaroscopy research.

## Supplementary Material

keaa723_Supplementary_Data

## References

[1] Matucci-Cerinic M , Allanore Y, Czirják L et al The challenge of early systemic sclerosis for the EULAR Scleroderma Trial and Research Group (EUSTAR) community. It is time to cut the Gordian knot and develop a prevention or rescue strategy. Ann Rheum Dis 2009;68:1377–80. 19674983 10.1136/ard.2008.106302

[2] Avouac J , Fransen J, Walker UA et al Preliminary criteria for the very early diagnosis of systemic sclerosis: results of a Delphi Consensus Study from EULAR Scleroderma Trials and Research Group. Ann Rheum Dis 2011;70:476–81. 21081523 10.1136/ard.2010.136929

[3] Koenig M , Joyal F, Fritzler MJ, Roussin A et al Autoantibodies and microvascular damage are independent predictive factors for the progression of Raynaud’s phenomenon to systemic sclerosis. A twenty-year prospective study of 586 patients, with validation of proposed criteria for early systemic sclerosis. Arthritis Rheum 2008;58:3902–12. 19035499 10.1002/art.24038

[4] Van den Hoogen F , Khanna D, Fransen J et al 2013 classification criteria for systemic sclerosis: an American College of Rheumatology/European League Against Rheumatism collaborative initiative. Arthritis Rheum 2013;65:2737–47. 24122180 10.1002/art.38098 PMC3930146

[5] Van den Hoogen F , Khanna D, Fransen J et al 2013 classification criteria for systemic sclerosis: an American College of Rheumatology/European League Against Rheumatism collaborative initiative. Ann Rheum Dis 2013;72:1747–55. 24092682 10.1136/annrheumdis-2013-204424

[6] Smith V , Herrick AL, Ingegnoli F et al Standardisation of nailfold capillaroscopy for the assessment of patients with Raynaud’s phenomenon and systemic sclerosis. Autoimmun Rev 2020;19:102458. 31927087 10.1016/j.autrev.2020.102458

[7] Ingegnoli F , Ughi N, Dinsdale G et al An international SUrvey on non-iNvaSive tecHniques to assess the mIcrocirculation in patients with RayNaud’s phEnomenon (SUNSHINE survey). Rheumatology Int 2017;37:1879–90. 10.1007/s00296-017-3808-0 28894946

[8] Hughes M , Moore T, O’Leary N et al A study comparing videocapillaroscopy and dermoscopy in the assessment of nailfold capillaries in patients with systemic sclerosis-spectrum disorders. Rheumatology 2015;54:1435–42. 25749623 10.1093/rheumatology/keu533

[9] Dinsdale G , Peytrignet S, Moore T et al The assessment of nailfold capillaries: comparison of dermoscopy and nailfold videocapillaroscopy (Letter). Rheumatology 2018;57:1115–6. 29447411 10.1093/rheumatology/key018

[10] Berks M , Tresadern P, Dinsdale G et al An automated system for detecting and measuring nailfold capillaries. Med Image Comput Comput Assist Interv 2014;17:658–65. 25333175 10.1007/978-3-319-10404-1_82 PMC4936512

[11] Berks M , Dinsdale G, Murray A et al Automated structure and flow measurement—a promising tool in nailfold capillaroscopy. Microvascular Res 2018;118:173–7. 10.1016/j.mvr.2018.03.016 PMC5956308 29605552

[12] Pickup LC , Capel DP, Roberts SJ, Zisserman A. Bayesian methods for image super-resolution. Comput J 2008;52:101–13.

[13] Cutolo M , Sulli A, Smith V. How to perform and interpret capillaroscopy. Best Practice Res Clin Rheumatol 2013;27:237–48. 10.1016/j.berh.2013.03.001 23731933

[14] Avouac J , Lepri G, Smith V et al Sequential nailfold videocapillaroscopy examinations have responsiveness to detect organ progression in systemic sclerosis. Semin Arthritis Rheum 2017;47:86–94. 28291582 10.1016/j.semarthrit.2017.02.006

[15] Paxton D , Pauling JD. Does nailfold capillaroscopy help predict future outcomes in systemic sclerosis? A systematic literature review. Semin Arthritis Rheum 2018;48:482–94. 29602558 10.1016/j.semarthrit.2018.02.005

[16] Weiss K , Khoshgoftaar TM, Wang D. A survey of transfer learning. J Big Data 2016;3:9.

[17] Dinsdale G , Roberts C, Moore T et al Nailfold capillaroscopy—how many fingers should be examined to detect abnormality? Rheumatology 2019;58:284–8. 30247696 10.1093/rheumatology/key293

[18] Snow MH , Saketkoo LA, Frech TM et al Early results from an American pilot survey among Scleroderma Clinical Trials Consortium Members on capillaroscopy use and how to best implement nailfold capillaroscopy training. Clin Exp Rheumatol 2019;37(Suppl 119):151. 31025929

[19] Bhakuni DS , Vasdev V, Garg MK et al Nailfold capillaroscopy by digital microscope in an Indian population with systemic sclerosis. Int J Rheum Dis 2012;15:95–101. 22324952 10.1111/j.1756-185X.2011.01699.x

[20] Parker MJ , Oliffe MT, McGill NW. An evaluation of two novel capillaroscopy techniques in suspected scleroderma-spectrum disorders: a single-centre cross-sectional study. Modern Rheumatol 2018;28:676–80. 10.1080/14397595.2017.1404179 29260602

