# Supplementary figures and images for: Comparison between low cost USB nailfold capillaroscopy and videocapillaroscopy: a pilot study
Source: Rheumatology (Oxford). 2020 Nov 24;60(8):3862–7. doi: 10.1093/rheumatology/keaa723 (PMC8328506; doi:10.1093/rheumatology/keaa723)

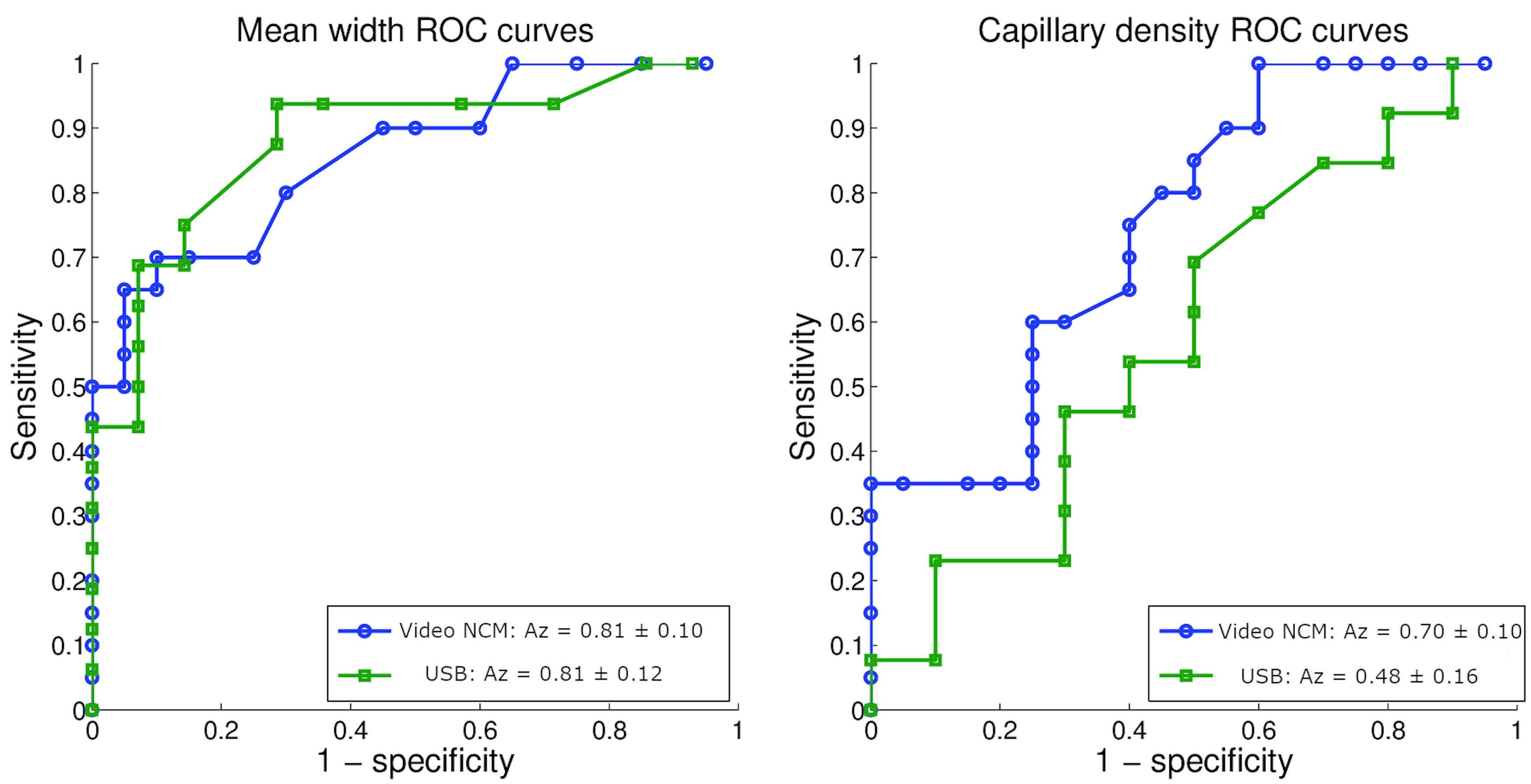

Supplement: keaa723_Supplementary_Data [file keaa723_supplementary_data.zip › rhe-20-1424-File004.tif]
